# Marketing and semiotic approach on communication. Consequences on knowledge of target-audiences

**Published:** 2014

**Authors:** Borţun Dumitru, Purcărea Victor Lorin

**Affiliations:** * National School of Political and Administrative Sciences SNSPA; ** Bucharest; “Carol Davila” University of Medicine and Pharmacy, Bucharest

**Abstract**

**The marketing approach**

In reply to the question “What is marketing?”, Kotler and Armstrong wrote: “More than any other company function, marketing deals with clients. Creating value and satisfaction for the client represents in itself the essence of modern marketing thinking and practice” (Kotler &Armstrong, *Principles of Marketing*, 2nd edition, Teora, Bucharest, 2003, p. 4). However, this does not mean that the only purpose of marketing is client satisfaction, regardless of the manufacturer’s expenses. In the same place, Kotler and Armstrong offered the simplest definition of marketing: “marketing is the delivery of client satisfaction at a profit.” (id).

Therefore, modern marketing places the *consumer* in the centre of attention, and not the producer, aiming at producing and selling *what* the consumer wants, *when* and *where* he wants it, *at a price he* is willing to pay. However, marketing is not only about “explaining and selling”; sale and advertising is only the tip of the iceberg. Unlike sales, which only begins after the product is manufactured, “marketing begins long before the company has a product” (*ibid*, p. 5).

In *Principles of Marketing*, Kotler and Armstrong define marketing as it follows: “a social and entrepreneurial process, through which individuals and groups obtain the thing they need and they desire, by creating and exchanging products and value with other groups and people” (id). It is a *social process* – an extremely complex process, and sale is, like advertising, only one of its many functions.

In a classic paper, *Introduction to Communication Sciences*, John Fiske showed that in the semiotic approach the message is a *construction of signs, which, by interacting with the receptor, generates meaning*. The focus is not so much on communication as process, but rather on communication as generator of meaning. The sender (message transmitter) loses his importance. The focus is directed towards to the “text” and the way it is “read”. “The reading” is the process of discovering the meaning that emerges when the “reader” interacts or *negotiates* with the “text”. The negotiation takes place when the “reader” filters the message through the strainer of the cultural pattern, in terms of signs and codes that make up the message. The more we share the same codes and the same sign system, the closer the two significances attributed to the message (John Fiske, *Introduction to Communication Sciences* Polirom, Iaşi, 2003, p. 61).

When we think in the terms of semiotic approach, which highlights constantly, biunique interactions between the message “producer” and the reference system, between him and the “reader”, we deal with the cultural determinism of communication, using the concepts of Kuhn and Gonseth (*paradigm* and *referential* respectively).

The concept of “cultural paradigm” has been used increasingly more over the last four decades, both in social philosophy, as well as in anthropology, psychology and sociology . It entered these fields by way of “concept translation”, borrowed from the philosophy of science, where it was imposed by the American philosopher Thomas S. Kuhn. He was the one to realize that theories on the nature of science and the purpose of research in natural sciences do not concord with the scientific practice, as it ensues from the history of science. In practice, he says, the behavior of scientists deviates from the canons that define *scientificity* and even *rationality* (canon which we encounter both in science philosophy and in current mentality).

The only realistic solution is to ***use tacit communication***, which could reach for the “self-image” of groups and individuals from various social groups, in order to trigger the change of some of the current cultural paradigm’s presuppositions, in particular of those who generate perceptions, representations and *value-attitude* couples, which, in their turn, generate contra-productive behaviors (which oppose the purposes of modernization). In essence, it is the art of *talking about something while leaving the impression that you talk about something else*. 

To those who will cry resentfully “This is manipulation!” we reply: 1) manipulation “abhors vacuum”, because if we do not manipulate, others will; 2) assuming nevertheless that no one is manipulating them, people would manipulate themselves- which they do, “day after day, hour after hour, and in mass”, by virtue of desiderative thinking, of inauthentic thinking (Erich Fromm) and of the “voluptuousness of self-deception” (Jean-Francois Revel); 3) manipulation is not evil in itself; it can be good or bad, depending on the purpose; 4) nothing great can be accomplished without manipulation – from bringing up a child to educating a people -, and we do not speak of the *modernization* of the Romanians, a much more complex and difficult task than education.

**Conclusion:** he who finds himself from manipulation is manipulating himself, but against him; he remains a «part of the problem and will never be a “part of the solution”».

A pre-condition for success is the positive knowledge of *value-attitude* couples that are at work in today’s Romanian society, knowledge that will be ensured by mixing theoretical approaches with national-scale sociologic research, carried out by a specialized institute.

**Keywords:** marketing and semiotic approach, communication, target audiences

